# Impact of the 12-gene recurrence score in influencing adjuvant chemotherapy prescription in mismatch repair proficient stage II/III colonic carcinoma—a systematic review and meta-analysis

**DOI:** 10.1007/s00384-023-04364-2

**Published:** 2023-03-13

**Authors:** Matthew G. Davey, Maeve O’Neill, Mark Regan, Babak Meshkat, Emmeline Nugent, Myles Joyce, Aisling M. Hogan

**Affiliations:** Department of Surgery, Galway University Hospitals, Galway, H91 YRY71 Ireland

**Keywords:** Colon cancer, Cancer genomics, Personalised medicine, Adjuvant therapies

## Abstract

**Introduction:**

The 12-gene recurrence score (RS) is a clinically validated assay which predicts recurrence risk in patients with stage II/III colon cancer. Decisions regarding adjuvant chemotherapy may be guided using this assay or based on the judgement of tumour board.

**Aims:**

To assess the concordance between the RS and MDT decisions regarding adjuvant chemotherapy in colon cancer.

**Methods:**

A systematic review was performed in accordance with PRISMA guidelines. Meta-analyses were performed using the Mantel–Haenszel method using the Review Manager version 5.4 software.

**Results:**

Four studies including 855 patients with a mean age of 68 years (range: 25–90 years) met inclusion criteria. Overall, 79.2% had stage II disease (677/855) and 20.8% had stage III disease (178/855). For the entire cohort, concordant results between the 12-gene assay and MDT were more likely than discordant (odds ratio (OR): 0.38, 95% confidence interval (CI): 0.25–0.56, *P* < 0.001). Patients were more likely to have chemotherapy omitted than escalated when using the RS (OR: 9.76, 95% CI: 6.72–14.18, *P* < 0.001). For those with stage II disease, concordant results between the 12-gene assay and MDT were more likely than discordant (OR: 0.30, 95% CI: 0.17–0.53, *P* < 0.001). In stage II disease, patients were more likely to have chemotherapy omitted than escalated when using the RS (OR: 7.39, 95% CI: 4.85–11.26, *P* < 0.001).

**Conclusions:**

The use of the 12-gene signature refutes the decision of tumour board in 25% of cases, with 75% of discordant decisions resulting in omission of adjuvant chemotherapy. Therefore, it is possible that a proportion of such patients are being overtreated when relying on tumour board decisions alone.

## Introduction

Adjuvant chemotherapy confers a survival advantage for patients treated for high-risk stage II and all patients with stage III colonic carcinoma [1–4]. Accordingly, these therapies have become embedded into the clinical guidelines and expert consensus statements [5, 6]. Nevertheless, almost 80% of patients diagnosed with stage II colonic cancers are cured by surgical resection alone, with limited survival advantage conferred by adjuvant chemotherapy [2], questioning therapeutic benefit. Notwithstanding these favourable oncological outcomes, the evidence supporting addition of 5-fluorouracil (5-FU)-based regimens in the adjuvant setting is controversial due to inconsistent results [7, 8]. Recent recommendations from American Society of Clinical Oncology (ASCO) endorse adjuvant chemotherapy use in ‘high risk’ stage II disease, with clinicopathological parameters used to inform tumour board regarding adjuvant chemotherapy [5]. In the setting of stage III disease, there remains debate as to the benefit of oxaliplatin and recommended duration [9–11], with adverse clinicopathological features used to guide its use, as in stage II disease [12]. Recently, International Duration Evaluation of Adjuvant Chemotherapy (IDEA) performed a pooled analysis of 6 randomised clinical trials, encompassing 12,835 patients with stage III disease. This illustrated the non-inferiority of shorter course of adjuvant chemotherapies (compared to longer courses), with premise to omit oxaliplatin in certain incidences [9]. Accordingly, these results have been incorporated into the National Comprehensive Cancer Network (NCCN) guidelines for managing stage III colon cancer [13].

There is however data highlighting inconsistencies in the reproducibility of several histopathological and molecular parameters to estimate recurrence risk in colon cancer [14, 15], questioning validity of adjuvant chemotherapy regimens. Translation research efforts have focused on identifying novel and reproducible biomarkers which estimate patient-specific risk of disease recurrence and inherent benefit of systemic chemotherapy.

Using formalin-fixed, paraffin-embedded resected specimens from four independent patient cohorts [16–19], O’Connell et al. designed and prospectively validated a multigene assay capable of estimating prognoses for patients with stage II/III colonic carcinoma [20]. This molecular signature, known as the 12-gene expression assay (commercially available as the OncotypeDX^©^ Recurrence Score (RS) from Genomic Health Inc.^©^, Redwood City, CA, USA) accurately provides prognostication and estimates derived benefit from chemotherapy [20]. This algorithm uses the reverse transcriptase polymerase chain reaction (RT-PCR) products of 7 cancer-related genes and 5 reference genes, producing a RS which represents the patient-specific risk of recurrence at long-term follow-up [20]. The 12-gene expression assay has subsequently been validated on several occasions [21–24], however is yet to be endorsed by expert consensus statements, recommendations or guidelines [25].

At present, individualising therapeutic strategies presents a challenge to tumour board [26]. This challenge, coupled with the potential of the 12-gene assay (and the inherent success of other RS assays in other malignancies [27–29]), suggests the signature may be of benefit in tailoring chemotherapy regimens to the needs of each patient. Evaluating the rates of concordance between the 12-gene expression assay and colorectal tumour board decisions may be useful for management of prospective patients. Thus, the objective of this study was to perform a systematic review and meta-analysis assessing congruency between the RS assay and tumour board decisions regarding adjuvant chemotherapy in patients with stage II/III colon cancer. The secondary outcome involved the assessment of the impact of discordant decisions the multigene assay and tumour board and whether treatment was omitted or escalated.

## Methods

This systematic review and meta-analysis was performed in accordance to the Preferred Reporting Items for Systematic Reviews and Meta-Analyses (PRISMA) and MOOSE guidelines [30, 31]. Local institutional ethical review and approval was not required as this is a review of previously published data. Each author contributed to formulating the study protocol and this was published on the International Prospective Register of Systematic Reviews (PROSPERO).

### PICO

Using the PICO framework [32], the aspects the authors wished to address were as follows:

- Population – Patients with newly diagnosed stage II/III mismatch proficient colonic carcinoma who were aged 18 years or older without distant metastatic disease who underwent 12-gene RS testing (Genomic Health Inc., Redwood City, CA) performed on their resected colonic carcinoma specimen. All included patients had to have prior recommendations from tumour board in relation to whether the patient would benefit from adjuvant chemotherapy prescription.
- Intervention – Any patient in the selected group found to RS testing results which a concordant with tumour board decision.
- Comparison – Any patient in the selected group found to RS testing results which a discordant with the tumour board decision.
- Outcomes – The rate of concordance and discordance of adjuvant chemotherapy decision making based on tumour board decisions and the results of the 12-gene RS assay.

### Search strategy

An electronic search was performed of the PubMed, Embase, Cochrane and Science Direct databases for relevant studies. The final search was performed on the 29^th^ of September 2022. This search was performed by two independent reviewers (MGD and MON), using a predetermined search strategy that was designed by the senior authors (AMH and MJ). This search included the search terms: (Oncotype DX) and (colorectal cancer), which was linked by the Boolean operator ‘AND’. Included studies were limited to the English language and were not restricted by year of publication. All duplicate studies were manually removed, before titles were screened, and studies considered appropriate had their abstracts and/or full-text reviewed. Retrieved studies were reviewed to ensure inclusion criteria were met for one primary and secondary outcome at a minimum. In cases of discrepancies of opinion, a third author was asked to arbitrate (EN).

### Inclusion and exclusion criteria

Clinical studies comparing patients with stage II/III mismatch repair proficient disease who had previously received a recommendation from the tumour board in relation to whether adjuvant chemotherapy were included. Resected specimens subsequently underwent RS genomic testing. All studies included patients aged 18 years or greater at the time of colonic cancer diagnosis. Outcomes of interest included RS testing, clinicopathological data and the concordance between tumour board recommendation and RS results. Studies including patients with metastatic disease (stage IV colonic carcinoma) were excluded. Published abstracts from conference proceedings were excluded, as were case reports, case series reporting outcomes in five patients or less, and editorial articles. Studies providing data which could not be utilised for meta-analyses were excluded.

### Data extraction and quality assessment

The following data was extracted and collated from retrieved studies meeting inclusion criteria: (1) first author name, (2) year of publication, (3) study design, (4) country of origin, (5) number of patients diagnosed with CRC, (6) number of patients with RS testing results, (7) tumour board recommendations, (8) median age (and range) at diagnosis, (9) mean RS, (10) RS categorisation, and (11) clinicopathological data. Risk of bias and methodology quality assessment was performed in accordance to the Newcastle–Ottawa scale [33].

### Statistical analysis

Descriptive statistics were used to determine associations between tumour board recommendations and RS categories. Data was expressed as dichotomous or binary outcomes, reported as odds ratios (ORs) were expressed with 95% confidence intervals (CIs) following estimation using the Mantel–Haenszel method. Either fixed or random effects models were applied on the basis of whether significant heterogeneity (*I*^2^ > 50%) existed between studies included in the analysis. Statistical heterogeneity was determined using *I*^2^ statistics. All tests of significance were two-tailed with *P* < 0.050 indicating statistical significance. Descriptive statistics were performed using the Statistical Package for Social Sciences (SPSS) version 26 (International Business Machines Corporation, Armonk, New York). Meta-analysis was performed using Review Manager (RevMan), Version 5.4 (Nordic Cochrane Centre, Copenhagen, Denmark). MGD performed each statistical analyses with supervision of the senior author (AMH).

## Results

### Literature search

**Fig. 1:**
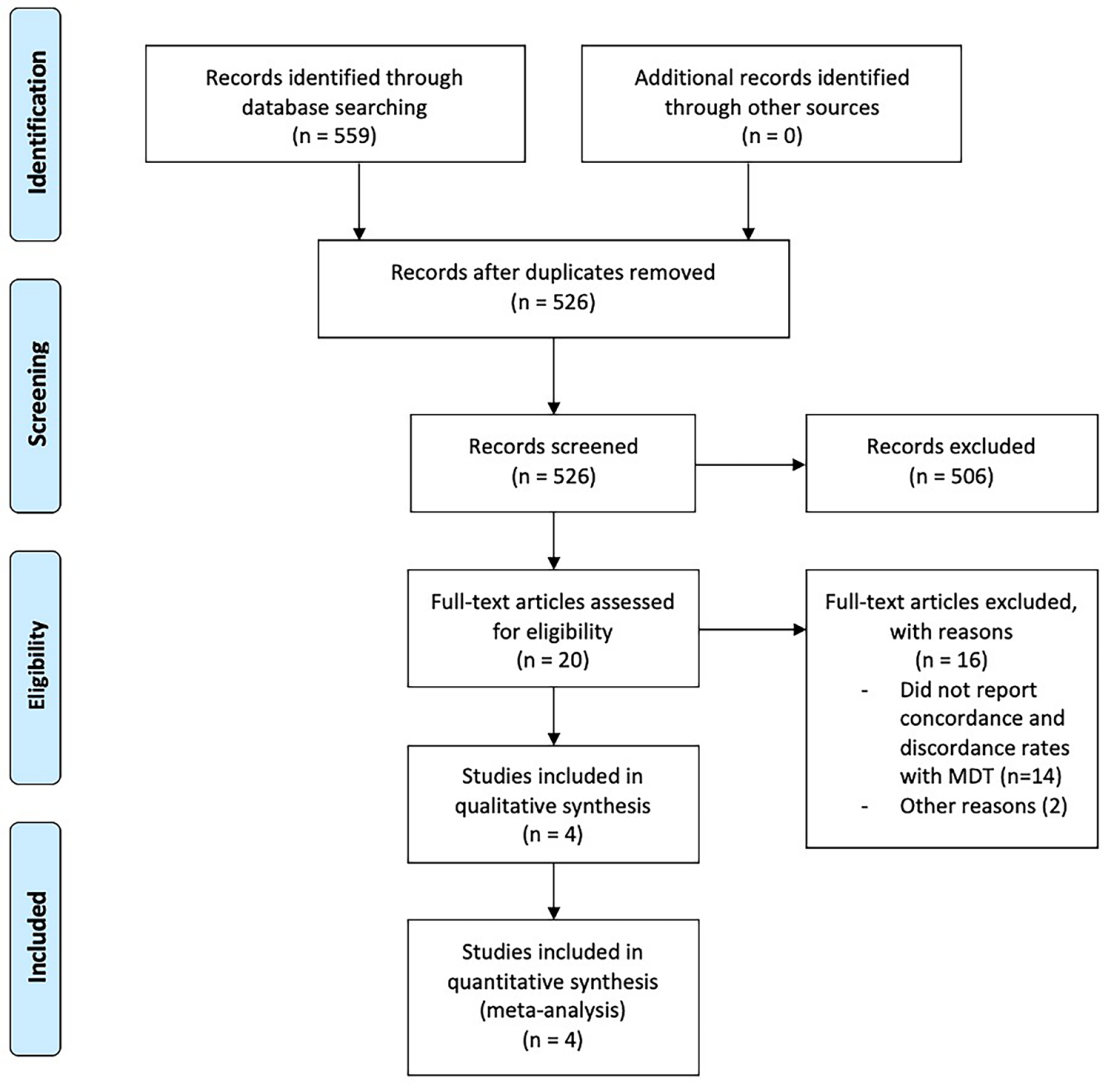
PRISMA flowchart illustrating the systematic search process

The initial electronic literature search retrieved 559 studies. Overall, 33 duplicate studies were manually extracted. The remaining 526 titles were screened for relevance, before 36 studies had their abstracts, and 20 manuscripts had their full texts reviewed. In total, 4 studies fulfilled the predetermined inclusion criteria and were included in this meta-analysis [34–37] (Table 1 and Fig. 1).

**Table 1:** Studies included in this systematic review and meta-analysis

Title	Year	Country	LOE	N	Mean age	Stage II	Stage III	NOS
Brenner	2015	Israel	Retrospective (III)	269	68 (60–75)	269	-	7
Cartwright	2014	USA	Retrospective (III)	92	-	92	-	6
Oki	2021	Japan	Prospective (II)	275	69 (25–90)	97	178	7
Srivastava	2014	USA	Prospective (II)	219	65 (27–87)	219	-	7
-	-	-	-	855	68 (25–90)	677	178	-

*LOE* level of evidence, *N* number, *NOS* Newcastle–Ottawa scale

### Study and patient characteristics

Four studies were included in this analysis, which included two prospective and two retrospective cohort studies respectively. Overall, 855 patients were included with a mean age of 68 years (range: 25–90 years). Overall, 79.2% of patients had stage II disease (677/855) and the 87.1% of patients had colonic cancers of the adenocarcinoma histological subtype (425/488). From the 3 studies reporting adjuvant chemotherapy prescription, 32.9% received adjuvant chemotherapy (164/499) and 67.1% underwent surveillance (335/499). Table 1 demonstrates patient demographic data and the risk of bias assessments for the individual studies included in this meta-analysis.

### 12-gene expression assay results

All 855 included patients underwent RS testing. The mean RS was 28 (range: 7–70). Of note, Brenner et al. was the sole study reporting mean RS results [34]. All studies used the traditional numerical categorisation of RS, which considered RS < 30 as low risk, RS 30–40 as intermediate risk, and RS > 40 as high risk as previously outlined [20]. Of those evaluated compared to the MDT treatment decisions, 71.7% had RS < 30 (556/776), 21.5% had RS 30–40 (167/776), and 6.8% had RS > 40 (53/776) (Table 2).

**Table 2:** Summary of 12-gene expression assay results for eligible patients and adjuvant chemotherapy prescription

Title	Year	*N*	RS ≤ **30**	RS 31–40	RS > 40	AC	Surveillance
Brenner	2015	269	157	85	27	75	194
Cartwright	2014	92	74	9	9	47	45
Oki	2021	275	225	40	10	-	-
Srivastava	2014	140	100	33	7	42	99
-	-	776	556	167	53	164	335

*N* number, *RS* recurrence score, *AC* adjuvant chemotherapy

### Overall changes to tumour board recommendations

**Fig. 2:**
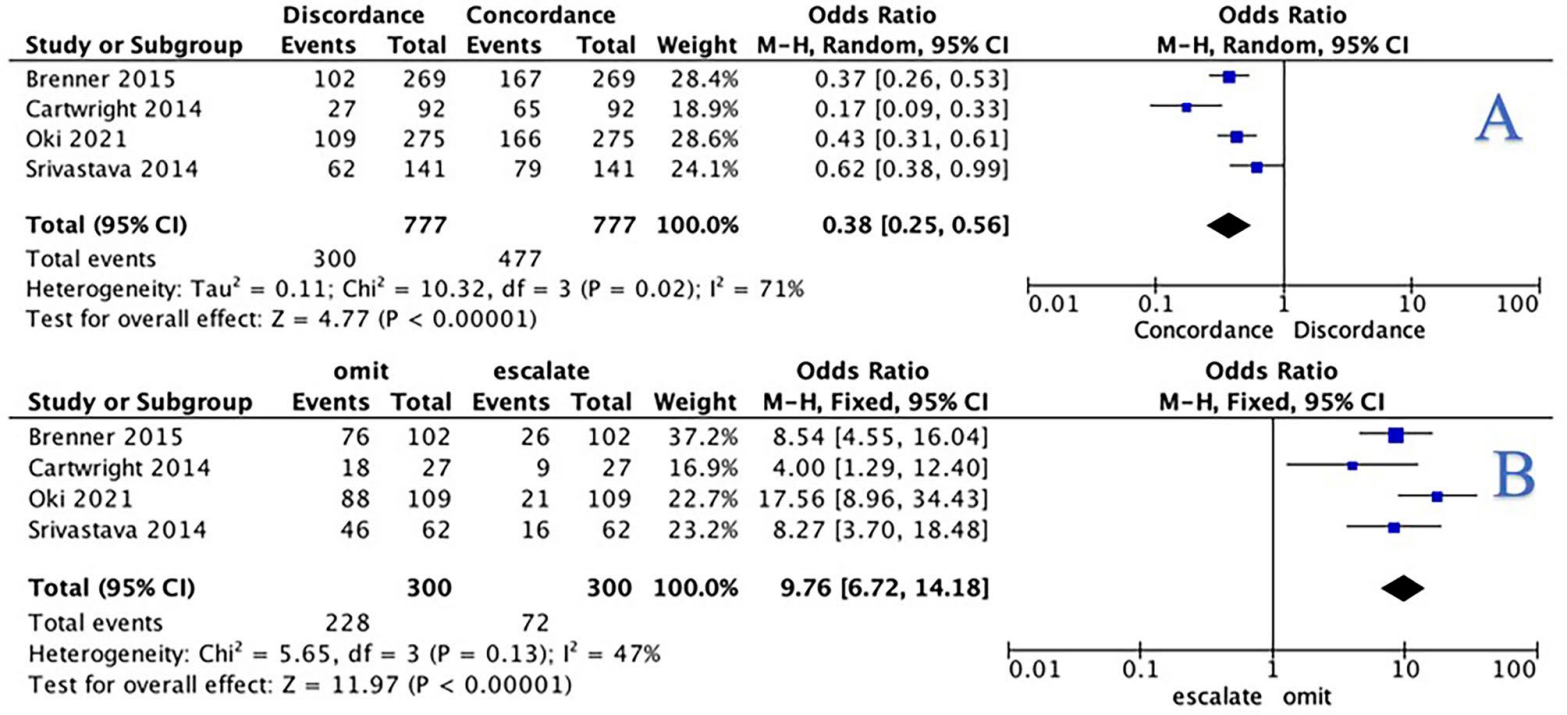
**A** Forest plot illustrating the likelihood of concordance or discordance between the 12-gene expression assay and the multidisciplinary team for the overall patient cohort. **B** Forest plot illustrating the likelihood of discordant 12-gene expression assay and the multidisciplinary team results leading to the omission or escalation of adjuvant chemotherapy prescription for the overall patient cohort

For the overall patient cohort, 25.8% of recommendations made by tumour board were discordant with the results of 12-gene expression assay testing (200/776) (Table 3). Concordant results between the 12-gene expression assay and tumour board were more likely than discordant results (odds ratio (OR): 0.38, 95% confidence interval (CI): 0.25–0.56, *P* < 0.001, heterogeneity (*I*^2^): 71%) (Fig. 2A). For patients with discordant results between the tumour board and the 12-gene expression assay, these were more likely to have adjuvant chemotherapy omitted (76.0%, 228/300) than treatment escalated to include adjuvant chemotherapy (or the addition of oxaliplatin) (24.0%, 72/300). At meta-analysis, these patients were almost 10 times more likely to have adjuvant chemotherapy omitted than escalated when relying on the 12-gene expression assay (OR: 9.76, 95% CI: 6.72–14.18, *P* < 0.001, *I*^2^ = 47%) (Fig. 2B).

**Table 3:** Impact of the 12-gene expression assay in changing multidisciplinary team decisions in relation to adjuvant chemotherapy on stage II/III colon cancer

Title	Year	Changes in MDT decision overall	Changes in MDT decision—stage II	Changes in MDT decision—stage III
Brenner	2015	102/269	102/269	-
Cartwright	2014	27/92	27/92	-
Oki	2021	109/275	29/97	80/178
Srivastava	2014	62/141	62/141	-
-	-	200/777	120/599	80/178

*MDT* multidisciplinary team

### Changes to tumour board recommendations for stage II disease

**Fig. 3:**
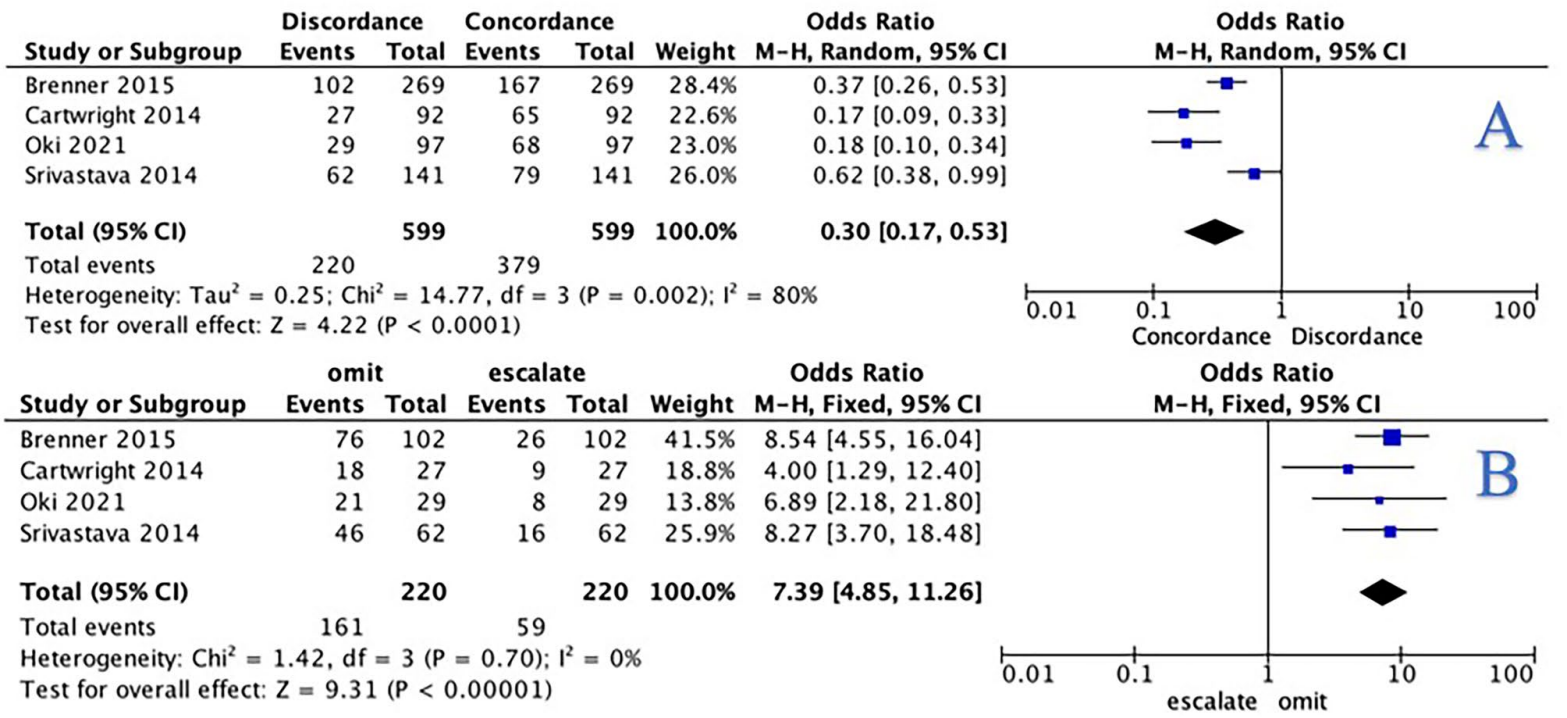
**A** Forest plot illustrating the likelihood of concordance or discordance between the 12-gene expression assay and the multidisciplinary team for those with stage II colon cancers. **B** Forest plot illustrating the likelihood of discordant 12-gene expression assay and the multidisciplinary team results leading to the omission or escalation of adjuvant chemotherapy prescription for those with stage II colon cancers

For patients with stage II disease, 20.0% of recommendations made by tumour board were discordant with the results of 12-gene expression assay testing (120/599) (Table 3). Concordant results between the 12-gene expression assay and tumour board were more likely than discordance (OR: 0.30, 95% CI: 0.17–0.53, *P* < 0.001, I^2^: 80%) (Fig. 3A). Patients with stage II disease with discordant tumour board and 12-gene expression assay results were more likely to have adjuvant chemotherapy omitted (73.2%, 161/220) than escalated to include adjuvant chemotherapy (26.8%, 59/220). At meta-analysis, patients with stage II disease were more than 7 times more likely to have adjuvant chemotherapy omitted than escalated when relying on the 12-gene expression assay (OR: 7.39, 95% CI: 4.85–11.26, *P* < 0.001, *I*^2^ = 0%) (Fig. 3B).

### Changes to tumour board recommendations for stage III disease

Discordance between recommendations made by tumour board and 12-gene expression assay testing results were more likely to occur in stage III disease (44.9%, 80/178) than in stage II disease (20.0%, 120/599) (*P* < 0.001) (Table 3). As results for stage III disease were only available from the study performed by Oki et al., meta-analysis could not be performed [37].

## Discussion

This is the first systematic review and meta-analysis evaluating the importance of using the 12-gene expression assay to guide adjuvant chemotherapy prescription in patients with resected stage II/III colonic carcinoma. The results illustrate that in a quarter of cases, the 12-gene assay provides treatment decisions discordant with that of the MDT, with treatment de-escalation more likely when the RS assay is used. Overall, these results suggest there is a proportion of patients with colonic carcinoma which may be currently overtreated with systemic chemotherapy, which may become more apparent when translating these results from the 855 patients included in this study to a population or global level. Notwithstanding these important findings, further prospective validation of the 12-gene expression assay’s utility is important to ensure oncological outcomes will not be jeopardised by the omission of adjuvant chemotherapy in this cohort, should the assay be used as the sole biomarker used to guide treatment decisions in contemporary colon cancer management.

In this study, an overall discordance rate of 25.8% in treatment decisions between the 12-gene expression assay and tumour board was observed. Discordance between the multigene signature was increasingly prominent in those with stage III disease (44.9%), compared to those with stage II disease (20.0%) (*P* < 0.001). This is an important finding, as the current management paradigm for colon carcinoma relies on objective assessment of histopathological features, such as lymphovascular invasion, differentiation, and microsatellite instability, to guide therapeutic decision making surrounding adjuvant chemotherapy [7]. Moreover, these results suggest the 12-gene assay captures important predictive biomolecular parameters which are not at the routine discretion of tumour board, when the assay is not available. Accordingly, this suggests the multigene expression assay may correctly identify patients who may be safely spared adjuvant treatment, due to limited long-term benefit of such therapies, and surpasses other risk stratification strategies: Recent data from the IDEA’s ACCENT database highlighted the failure of the IDEA risk classification in successfully predicting the benefit of the addition of oxaliplatin to conventional chemotherapy in stage III colon cancer [38]. Thus, the current analysis highlights the pragmatism of the RS assay to facilitate a personalised approach to oxaliplatin chemotherapy in the setting of stage III colonic carcinoma.

Adjuvant chemotherapy regimens were de-escalated in 75% of cases where 12-gene expression assay refuted decisions of tumour board, with 73% of those with stage II disease having adjuvant chemotherapy omitted completely. Therefore, this suggests the 12-gene signature facilitates judicious use of adjuvant chemotherapy, as for every three patients spared treatment, one patient will require having their treatment escalated. These findings support the recent work of Fu et al.. In their analysis of 58,133 patients with stage II colon cancer, the authors observed inferior oncological outcomes for those in receipt of adjuvant chemotherapy when analysing data from the Surveillance, Epidemiology, and End Results (SEER) database over a 25-year period (1988–2013) [39]. The modern perception is that the omission of adjuvant chemotherapy in the setting of stage II colon cancer may be acceptable for a majority [5]. This could suggest that the utility of molecular signatures (such as RS) in the setting of stage II disease may be questionable, given that a majority could be spared systemic treatment. Nevertheless, there remains a subgroup of patients with RS > 40 who do benefit from adjuvant chemotherapy [20], validating the requirement of such biomarkers in tailoring treatment bespoke. This analysis illustrates the role of the RS in refuting the decisions of four independent tumour boards, across three continents, indicating that RS assay provides a degree of consistency which may not be congruent across the globe [40]. Furthermore, this becomes even more evident when considering those diagnosed with stage III colon cancer, where patient selection using the 12-gene expression assay refuted tumour board decisions in 45% of cases. Therefore, the routine use of multigene signatures may be considered useful in providing reproducible results while individualising patient care in the era where personalised medicine is paramount.

In this study, mean RS was 28 and approximately 70% of patients were classified as being ‘low-risk’, 20% classified as ‘intermediate-risk’ and less than 10% as ‘high-risk’ of recurrence, as stratified by the 12-gene assay. Overall, these results are largely in keeping with previous studies [41, 42], and integrates international data, including patients of various ethnic origins [34–37]. It is well described that socioeconomic, genetic and racial disparities are factors which may confound results within cancer research and genomic testing [43–45]; therefore, consideration for such confounding factors is imperative upon translating these results into clinical practice worldwide.

This study is subject to certain unavoidable limitations. Firstly, while the RS assay has become embedded into contemporary management of breast and prostate cancers, its adoption into the management of colonic carcinoma has been less robust, potentially limiting the impact of these results in clinical practice. Secondly, of the four included studies, just two were of retrospective design, inferring the inherent risks of ascertainment, selection and confounding biases [34, 35]. These factors inevitably and uncontrollably limit the results of this study. Thirdly, the current analysis only included 6.8% of patients classified as ‘high-risk’ (56/776). Although this is consistent with previously published data [41, 42], other analyses have previously illustrated a higher proportion of ‘high-risk’ patients, for which discordance rates with tumour board decisions are not quantified. Fourthly, it is important to note the direct and indirect conflict of interests reported between authors of all included studies and the Genomic Health Inc. who develop the RS assay. This inevitably limits the transparency and robustness of the results yielded in this study. Finally, and most importantly, this analysis fails evaluate the long-term oncological implications of the discordant decisions observed between the RS and tumour board; these results will require thorough interrogation in a prospective fashion prior to implementation as standard of care in conventional colonic carcinoma management. Despite these limitations, the authors wish to reiterate that this analysis is the largest providing real world data comparing outcomes following 12-gene expression assay characterisation and tumour board decisions regarding adjuvant chemotherapy in the setting of stage II/III colonic carcinoma.

In conclusion, this systematic review and meta-analysis of current evidence illustrates the value in the 12-gene expression assay to guide therapeutic decision making when compared to that of international tumour boards. This is evident as using this molecular signature refutes the treatment decisions of the tumour board in 25% of cases, with 75% of these discordant decisions resulting in the omission of adjuvant chemotherapy. Overall, these results suggest there is a proportion of patients with colonic carcinoma which may be currently overtreated with limited oncological benefit. Thus, further prospective validation of 12-gene expression assay in colon cancer is warranted to ensure the individualization of adjuvant chemotherapy decision making.
